# The Waiting Game: How NGS and Guideline-Concordant-Care Timing Shape Survival in Advanced NSCLC

**DOI:** 10.3390/cancers18142287

**Published:** 2026-07-16

**Authors:** Melina E. Marmarelis, Adrienne M. Gilligan, Tomoko Sugihara, Tyler Marquart, Sam Whipple, Yufei Wang, Taylor J. Allen-Coyle, Naleen Raj Bhandari, Charu Aggarwal

**Affiliations:** 1 Perelman Center for Advanced Medicine, Philadelphia, PA 19104, USA; 2 Eli Lilly and Company, Indianapolis, IN 46285, USA; 3 Syneos Health, Morrisville, NC 27560, USA

**Keywords:** next-generation sequencing, targeted therapy, NSCLC, real-world evidence, turnaround time, overall

## Abstract

**Background**: Next-generation sequencing (NGS) and the initiation of biomarker-driven first-line (1L) treatment on overall survival (OS) was evaluated in advanced/metastatic non-small-cell lung cancer (a/mNSCLC). By applying a time-dependent regression modeling approach accounting for fluidity of clinical care, this study aimed to capture the dynamic interplay between NGS testing, turnaround time (TaT), treatment decisions and timing of initiation, and outcomes more comprehensively than traditional static analyses. **Methods**: This study was a retrospective observational study using the US Flatiron Health EHR-derived de-identified database. Eligible adults had non-squamous a/mNSCLC and received blood-based (B-NGS) or tissue-based (T-NGS) NGS within 90 days of diagnosis (1 January 2018–30 June 2023). A time-dependent covariate captured the NGS TaT, presence of a targetable alteration, time to 1L initiation, and 1L regimen (targeted concordant vs. discordant). OS was measured from the NGS order date to death. **Results**: A total of 1806 patients underwent B-NGS and 2583 underwent T-NGS. Starting empiric discordant 1L therapy before NGS results was associated with higher mortality versus initiating targeted concordant therapy after biomarker identification: B-NGS hazard ratio (HR): 1.58 (95% CI, 1.10–2.26); T-NGS HR: 1.07 (0.82–1.39). Considering discordant 1L therapy irrespective of timing, mortality remained higher versus targeted concordant therapy: B-NGS HR: 1.35 (1.04–1.74); T-NGS HR: 1.24 (0.98–1.56). **Conclusions**: This study demonstrates that waiting for comprehensive molecular profiling results before starting 1L treatment enables the delivery of appropriate biomarker-driven therapy, which translates into improved patient outcomes. Delays in NGS result availability or treatment initiation were linked to worse survival, underscoring the need for streamlined diagnostic workflows and rapid testing modalities.

## Simple Summary

Around half of patients with a common type of advanced lung cancer have specific genomic changes in their tumors that can be treated with matched, targeted drugs. Finding these changes through comprehensive biomarker testing before starting treatment is critical for choosing the most effective therapy. By using de-identified patient records from a large US cancer database, this study examined whether waiting for genetic test results before starting treatment led to better survival outcomes among 4389 patients (1806 tested using blood samples and 2583 tested using tumor tissue samples). The findings show that patients whose treatment was guided by their test results, receiving a therapy matched to their tumor’s genomic profile, lived longer than those who started treatment without this information. Delays in receiving test results or beginning the appropriate therapy were linked to worse outcomes. These results highlight the need for faster testing processes and broader use of rapid testing methods so that patients can receive the most effective treatment as early as possible.

## 1. Introduction

The treatment landscape for advanced/metastatic non-small-cell lung cancer (a/mNSCLC) has evolved significantly and is now guided by identification of actionable biomarkers [1]. Approximately 50% of patients with non-squamous a/mNSCLC harbor an actionable biomarker with an FDA-approved targeted therapy [2,3]. Guidelines recommend broad molecular profiling for a/mNSCLC to identify key actionable biomarkers with FDA-approved targeted therapies (i.e., EGFR, ALK, BRAF, ROS1, MET, NTRK, RET, KRAS G12C, HER2 [ERBB2] and NRG1 gene fusion) [4,5]. Targeted therapies have demonstrated superior clinical outcomes compared to chemoimmunotherapy or chemotherapy in patients with biomarker-driven a/mNSCLC [6].

Next-generation sequencing (NGS) has revolutionized cancer genomics by enabling the simultaneous and comprehensive molecular profiling of tumors. NGS can be used to test patients’ tumor samples (tissue- or liquid-based) for the presence of potential biomarkers, ideally prior to initiating first-line (1L) treatment. Real-world data have characterized trends in NGS testing among patients with a/mNSCLC, including increased testing rates, reflecting a shift toward earlier testing aligned with the expansion of actionable molecular targets [7,8,9].

Turnaround times (TaTs) for NGS testing (the interval between the test order and receipt of results) can be lengthy and vary across sample types, laboratories, settings, and institutions. Given the aggressive nature of the disease, healthcare providers may choose to initiate 1L treatment before receiving test results, potentially compromising long-term outcomes. Depending on clinical feasibility, some providers may initiate chemotherapy- or immunotherapy-based regimens to stabilize disease while awaiting molecular test results, with the intent to transition to targeted therapy if a biomarker is identified, whereas others may delay treatment initiation to wait for NGS. While prior studies have consistently shown that delays in treatment initiation are associated with worse overall survival (OS), the critical question is whether prolonged TaTs from NGS testing contribute to these delays. Availability of molecular genotyping results before 1L therapy was shown to be associated with significantly better OS compared with those with unavailable testing [10]. Concurrent tissue and plasma testing was associated with a greater likelihood of result availability before 1L therapy compared to tissue testing alone, as the addition of plasma testing increased the detection of therapeutically targetable mutations from 20.5% (tissue alone) to 35.8% [11].

Most retrospective evaluations of NGS TaTs have relied on static analytic approaches that consider treatment assignment as a fixed event [6,10,12,13,14]. Such methods fail to account for the timing of test results and therapy initiation, which can vary substantially in real-world practice, and therefore risk introducing immortal time bias and overstating the benefit of biomarker-driven therapy [15,16,17,18]. Time-dependent methods, such as Cox models with time-varying covariates or landmark analyses, address this limitation by incorporating when patients actually become eligible for and receive targeted therapy, yielding more valid and clinically relevant estimates [18,19]. Another challenge in prior retrospective studies is limited generalizability: many analyses include only patients who ultimately received therapy, excluding those who were tested but died before treatment initiation [20,21]. By applying time-dependent covariates, this study aimed to capture the dynamic interplay between NGS testing, TaT, treatment decisions and timing of initiation, and patient outcomes more comprehensively than traditional static analyses.

Therefore, the primary objective of this study was to evaluate OS in patients with a/mNSCLC by measuring the impact of waiting for NGS results before initiating 1L therapy. Specifically, we compared OS across two scenarios: (a) one including those who waited for NGS results and initiated first-line discordant therapy versus those who waited and initiated concordant targeted therapy; and (b) another including those who started first-line discordant therapy without waiting for NGS results versus those who waited and initiated concordant targeted therapy. Time-dependent factors, such as the availability of the NGS test result, biomarker status, time to initiation of 1L treatment, and type of 1L treatment, were used to better model routine clinical practice. The secondary objective evaluated the independent association of TaT for NGS with OS across the overall population by controlling for several covariates. Finally, as a tertiary objective, we sought to identify whether there was an optimal TaT by establishing a threshold at which a prolonged TaT adversely impacts OS. The goal of this study was to elucidate the importance of conducting timely and comprehensive molecular testing at the time of diagnosis of a/mNSCLC.

## 2. Materials and Methods

### 2.1. Design and Database

This retrospective observational study used the Flatiron Health electronic health record (EHR)-derived de-identified database (FHRD) in the US to identify a longitudinal cohort of patients diagnosed with a/mNSCLC between 1 January 2018 and 30 June 2023, with a data cutoff of 31 March 2024. The database comprises de-identified patient-level structured and unstructured data, curated via technology-enabled abstraction across approximately 280 cancer clinics (~800 sites of care) [22].

### 2.2. Cohort Selection

Patients with a diagnosis of non-squamous a/mNSCLC, aged 18 years or older, who had received either a blood-based (B-NGS) or tissue-based (T-NGS) NGS test within 90 days after a/mNSCLC diagnosis were included. The B-NGS and T-NGS cohorts were mutually exclusive in this analysis. Patients were classified according to the first NGS test type received within 90 days of a/mNSCLC diagnosis. Patients with records indicating more than one NGS test following diagnosis were excluded to minimize exposure misclassification and ensure non-overlapping testing cohorts.

Exclusion criteria included patients who had an NGS specimen collection date or NGS specimen receipt date prior to a/mNSCLC diagnosis; had a missing NGS specimen collection date; received single-gene tests for any biomarker prior to the NGS test result date; had an unknown biomarker test type prior to the NGS specimen collection date; had the NGS specimen receipt date prior to the NGS specimen collection date; had records for >1 NGS test following a/mNSCLC diagnosis; received 1L therapy prior to the NGS specimen collection date; received an investigational drug (masked in the database) as a 1L treatment; or received 1L treatment regimens not FDA-approved for NSCLC.

### 2.3. Study Measures

#### 2.3.1. Outcome

The outcome was overall survival (OS), defined as either the time from the NGS test order date to any-cause death (primary objective and secondary objective, respectively) or the time from the NGS test result date to any-cause death (tertiary objective). The specimen collection date was considered a close proxy for the test order date, which is unavailable in the database. Patients alive at the end of the follow-up were censored at the last activity date, which was defined as the last observed recording of vital information, a medication administration, or a laboratory test/result being reported.

#### 2.3.2. Exposures of Interest

**Figure 1:**
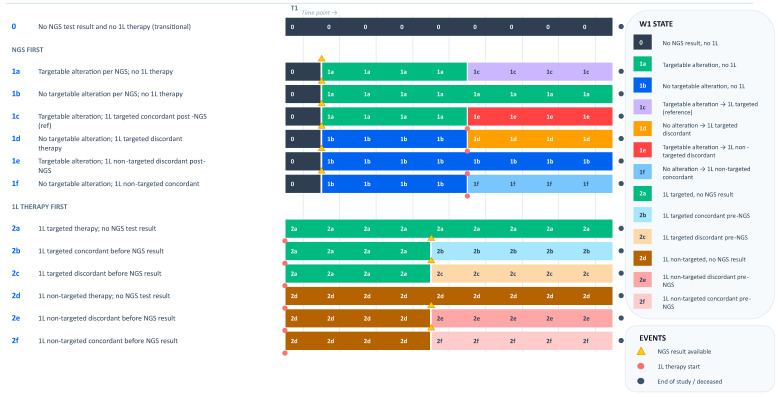
Time-dependent W1. This figure shows time-dependent changes in patient status during this study or until death. Each row represents a one-patient scenario defined by next-generation-sequencing (NGS) test results and intervention lines (1L). W1 = 0; scenarios a and b are transitional, non-final stages. Patients entering these stages may move to other stages.

For the primary objective, the exposure of interest was a blended time-dependent variable, W1 (Figure 1). W1 was defined by combining the following factors: NGS TaT (time from NGS test order date to result date); presence or absence of a targetable alteration (as determined by NGS testing); time from NGS test order date to initiation of 1L treatment relative to result availability; and type of 1L treatment (none, targeted, or non-targeted). Eligible biomarker-driven therapies included those targeting EGFR, ALK, BRAF V600E, ROS1, MET exon 14 skipping alteration, RET, NTRK, KRAS G12C, and HER2 (Table A1). The reference group (W1 = 1c) consisted of patients with a targetable alteration who began 1L biomarker-guided targeted therapy after receiving NGS test results—that is, patients who delayed initiation of first-line concordant targeted therapy until confirmation of biomarker-driven disease via NGS testing.

Two key comparator groups of interest were: (1) patients with a targetable alteration who subsequently initiated 1L non-targeted discordant therapy after the alteration was identified (W1 = 1e)—that is, patients who began 1L discordant therapy despite having confirmed biomarker-driven disease; and (2) patients who initiated 1L non-targeted discordant therapy prior to receiving NGS test results (W1 = 2e)—that is, patients who began 1L discordant therapy before being identified as having a targetable alteration.

For the secondary objective, the exposure of interest was a continuous time-dependent measure of the NGS TaT, W2, and consisted of the entire study population, stratified by sample type.

For the tertiary objective, the exposure of interest was the continuous distribution of the TaT and consisted of the entire study population, stratified by sample type.

### 2.4. Statistical Analysis

Characteristics at index (date of NGS test order or NGS test result) were summarized using descriptive statistics. Missing data were reported in the descriptive summary tables for their frequency count and percentage. OS was estimated using the Kaplan–Meier method, and unadjusted comparisons between groups were made using log-rank tests.

For the primary objective, a stepwise multivariable Cox proportional hazards model was used to compare OS among the W1 groups. Hazard ratios (HRs) and 95% confidence intervals (CIs) were estimated for each W1 category relative to the reference group. In this model, the total number of lines of therapy (LOTn) was included as a time-dependent covariate to account for the impact of subsequent treatment lines on OS.

For the secondary objective, a multivariable Cox model was fitted to evaluate the independent association between the continuous NGS TaT (W2) and OS. This analysis included the following time-dependent covariates: the LOTn, the NGS test result status (T2; no result, targetable alteration detected, or no targetable alteration detected), and the type of 1L treatment received (T3; none, targeted therapy, or non-targeted therapy). Time-dependent covariates were used to reduce immortal time bias by allocating person-time to testing and treatment states as they occurred rather than at baseline. This approach mitigates bias that may otherwise arise when patients must survive long enough to receive NGS results or initiate first-line therapy in observational analyses.

For the tertiary objective, the exposure of interest was the continuous distribution of the TaT, to which statistical criteria as detailed in Contal et al. and Williams et al. were applied to establish a threshold value of the NGS TaT (i.e., optimal cutpoint) that best separated low- and high-risk patients with respect to OS [23,24]. LOTn, W2, T2, and T3 were included as time-dependent variables.

All multivariable analyses were conducted, separately, for the B-NGS and T-NGS patient cohorts. Each Cox model also included baseline covariates (Appendix A). To examine whether these associations varied by baseline performance status, the multivariable models were additionally fitted within strata defined by ECOG performance status (0, 1, and 2+) for each cohort; stratum-specific estimates are presented in Appendix A Table A5 (B-NGS) and Table A6 (T-NGS). Missing data were modeled as a separate category without imputations or exclusions. All analyses were performed using SAS v9.4 (SAS Institute, Cary, NC, USA).

## 3. Results

### 3.1. Baseline Characteristics

**Figure A1:**
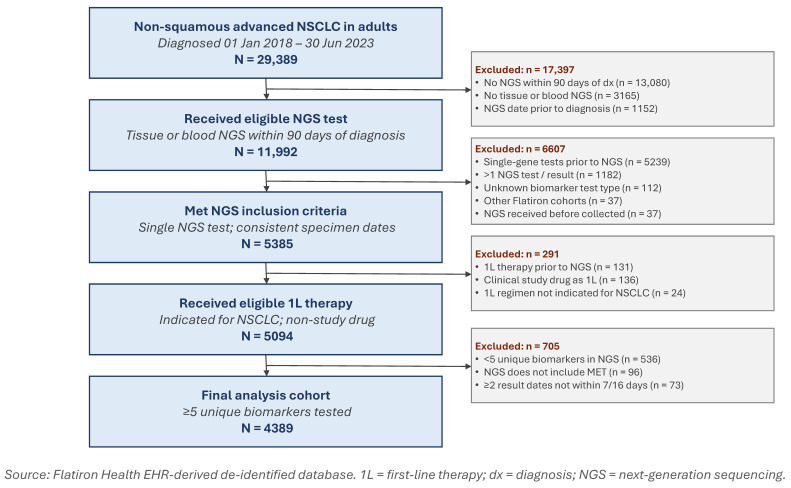
Study cohort selection. Single-gene tests: FISH, IHC (excluding IHC for PD-L1), mass spectrometry, other sequencing method (excluding Caris Life Sciences labs), PCR, and RNA sequencing (excluding Tempus/Caris Life Sciences labs). **Abbreviations**: a/mNSCLC, advanced/metastatic non-small-cell lung cancer; NGS, next-generation sequencing; 1L, first-line therapy; MET, Mesenchymal–Epithelial Transition factor.

A total of 4389 patients (1806 B-NGS and 2583 T-NGS) with non-squamous a/mNSCLC met all study eligibility criteria (Figure A1), with a median age of 71 (Q1 (first quartile), Q3 (third quartile): 63, 78) years, 52% female, 63% White, and 85% smokers (Table 1). Overall, approximately 78% of patients received 1L therapy, 17% died before receiving 1L therapy, and 5% had no evidence of 1L therapy during the study period. Among those that died before receiving 1L therapy, most were biomarker-negative (62%). Additional characteristics are presented in Table 1 and Table A2.

### 3.2. NGS Testing

The median (Q1, Q3) time from the diagnosis of a/mNSCLC to the NGS test order was 15 (8–26) days for B-NGS and 0 (0–0) days for T-NGS. The median (Q1, Q3) NGS TaT was 8 (7–11) days for B-NGS tests and 28 (21–41) days for T-NGS tests. In total, 37.8% of patients in the B-NGS cohort and 39.1% of patients in the T-NGS cohort were positive for a targetable alteration with available FDA-approved treatments (Table 1).

### 3.3. OS Based on Type of 1L Therapy After Waiting for NGS Test Result

**Figure 2:**
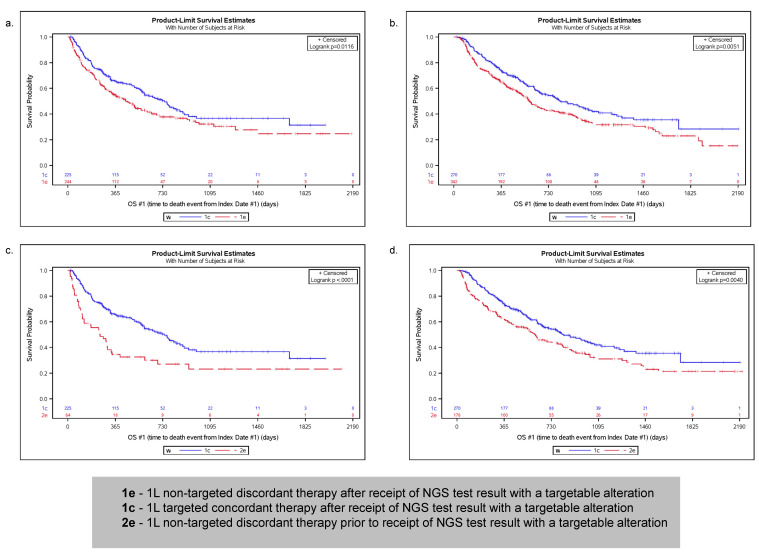
Unadjusted overall survival for patients in B-NGS and T-NGS cohorts. Overall survival probabilities for patients in (**a**) 1L non-targeted discordant therapy after receipt of NGS test result with a targetable alteration (1e) versus 1L targeted concordant therapy after receipt of NGS test result with a targetable alteration (1c) for B-NGS; (**b**) 1e versus 1c for T-NGS; (**c**) 1L non-targeted discordant therapy prior to receipt of NGS test result with a targetable alteration (2e) versus 1c for B-NGS; and (**d**) 2e versus 1c for T-NGS. Kaplan–Meier estimates of overall survival are shown for the following subgroup comparisons: 1e versus 1c represents patients who received 1L non-targeted discordant therapy versus patients who received 1L targeted concordant therapy after receipt of NGS test result, and 2e versus 1c represents patients who received 1L non-targeted discordant therapy and later had a positive NGS result versus patients who received 1L targeted concordant therapy after a positive NGS result. The x-axis represents time in days, with the number of individuals at risk displayed above the axis for each time interval, and the y-axis shows the survival probability.

Patients with a targetable alteration who initiated 1L targeted concordant therapy after receipt of their NGS test result experienced longer OS compared to those who initiated 1L non-targeted discordant therapy after receipt of their NGS test result. The unadjusted median (95% CI) OS was 24.6 (18.9–29.2) months versus 14.7 (11.1–18.7) months in the B-NGS cohort (*p* < 0.05; Figure 2a), while it was 27.2 (21.8–35.2) months versus 19.2 (17.1–22.4) months in the T-NGS cohort (*p* < 0.01; Figure 2b). Adjusted analysis demonstrated increased hazard of death among patients who initiated 1L non-targeted discordant therapy compared to those who initiated 1L targeted concordant therapy after being identified with biomarker-driven disease, which was statistically significant in B-NGS (hazard ratio (HR, 95% CI): 1.35 (1.04–1.74), *p* = 0.02) and nonsignificant in T-NGS (1.24 (0.98–1.56), *p* = 0.07; Table 2).

### 3.4. OS Based on Timing of Receipt of NGS Test Result and Type of 1L Therapy

Patients with a targetable alteration who initiated 1L targeted concordant therapy after receipt of their NGS test result experienced longer OS compared to those who initiated 1L non-targeted discordant therapy prior to receipt of their NGS test result indicating a targetable alteration. The unadjusted median (95% CI) OS was 24.6 (18.9–29.2) months versus 8.2 (3.7–11.0) months in the B-NGS cohort (*p* < 0.0001, Figure 2c), while it was 27.2 (21.8–35.2) months versus 20.0 (14.6–26.8) months in the T-NGS cohort (*p* < 0.01, Figure 2d). Adjusted analysis demonstrated increased hazard of death among patients who initiated 1L discordant therapy prior to waiting for their NGS test result compared to those who initiated 1L targeted concordant therapy after being identified with biomarker-driven disease, which was statistically significant in B-NGS (HR [95% CI]: 1.58 [1.10–2.26]) and nonsignificant in T-NGS (1.07 [0.82–1.39], *p* = 0.61; Table 2).

Adjusted HR (95% CI) estimates for other comparisons of W1 versus the reference are included in Table A3. Corresponding adjusted estimates within each ECOG performance status stratum (0, 1, and 2+) are presented in Appendix A Table A5 (B-NGS) and Table A6 (T-NGS).

### 3.5. Independent Association of NGS TaT with OS

In both B-NGS and T-NGS cohorts, the TaT for NGS testing was not independently associated with OS, including adjustment for several covariates (Table A4).

### 3.6. Optimal NGS TaT

**Figure A2:**
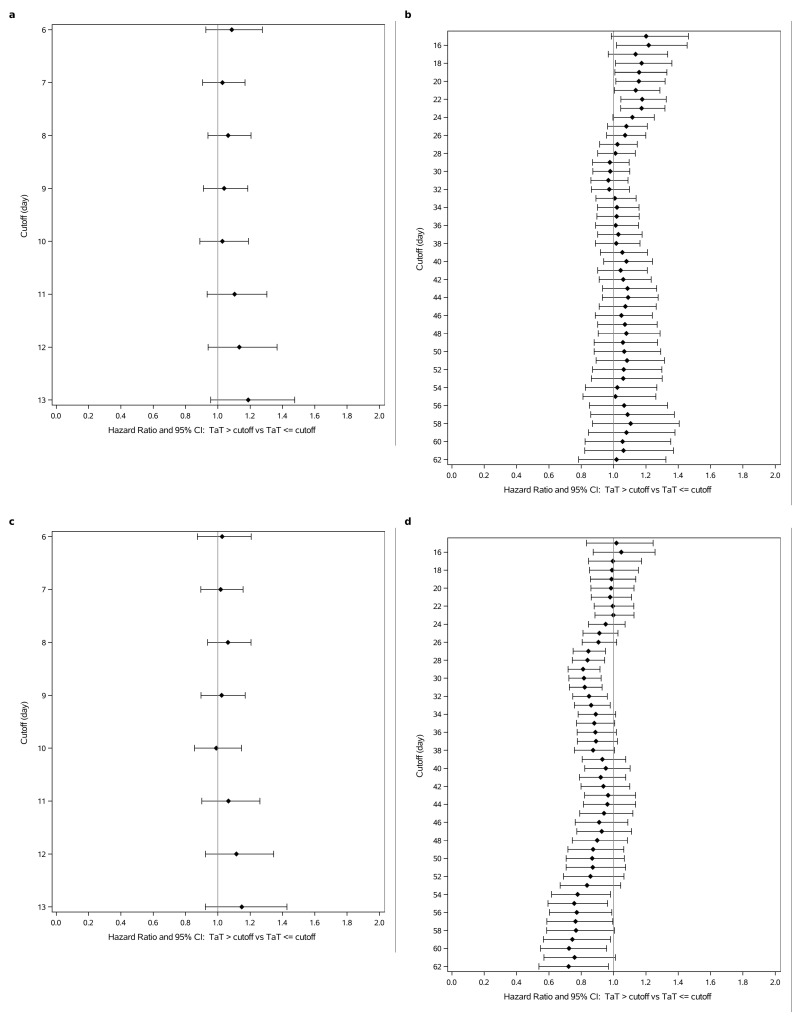
Turnaround time thresholds in B-NGS and T-NGS. (**a**) Unadjusted blood NGS TaT; (**b**) unadjusted tissue NGS TaT; (**c**) adjusted blood NGS TaT; (**d**) adjusted tissue NGS TaT.

While variability in the TaTs for NGS testing differed between the B-NGS and T-NGS cohorts, cutoff values for TaTs for NGS testing ranged from 23–29 days for T-NGS, with a trend suggesting that a longer TaT decreased hazard of death (Figure A2). No dichotomized comparisons of TaTs that best separated low- and high-risk patients with respect to OS met the Bonferroni-adjusted significance threshold (*p* < 0.0001).

## 4. Discussion

To the best of the authors’ knowledge, this is the first study to account for the fluidity of clinical care by evaluating several time-dependent factors that affect routine clinical practice including NGS and incorporating the role of the TaT, presence or absence of a targetable alteration, time to initiation of 1L treatment, and type of 1L treatment. Findings demonstrate that waiting for comprehensive molecular profiling results before starting 1L treatment enables the delivery of appropriate biomarker-driven therapy, which translates into improved patient outcomes. Among patients with biomarker-driven a/mNSCLC, receiving 1L targeted concordant therapy immediately after a positive NGS result was associated with substantially longer OS compared to receiving non-targeted therapy, which may have been initiated empirically or due to misinterpretation of biomarker results. This suggests that the survival benefit of targeted agents may not be fully recoverable when initiation is delayed. These results reinforce the need for streamlined testing workflows, reflex ordering, and rapid turnaround strategies—such as plasma-based NGS—to ensure that actionable results are available before treatment decisions, thereby maximizing the therapeutic window for patients with biomarker-driven disease.

For patients in the T-NGS cohort, the NGS specimen collection date is recorded as the date of diagnostic tissue biopsy, which is contemporaneous with or immediately following the a/mNSCLC diagnosis date in the Flatiron database. This reflects the common practice of reflex NGS ordering at the time of biopsy at participating sites, resulting in a median time from a/mNSCLC diagnosis to T-NGS specimen collection of 0 (IQR: 0–0) days. This should not be interpreted as a causal delay of zero but rather as an artifact of the database structure. The B-NGS and T-NGS cohorts are mutually exclusive; patients with records of more than one NGS test following diagnosis were excluded. The longer median time from diagnosis to B-NGS specimen collection (15 days; IQR: 8–26) is consistent with the use of B-NGS predominantly as a sequential or reflex strategy following initial tissue-based evaluation in this cohort.

An important practical implication of these findings concerns the role of B-NGS as a strategy to compress TaTs and enable earlier biomarker-driven treatment decisions. In this cohort, the median B-NGS TaT was 8 days (IQR: 7–11), compared with 28 days (IQR: 21–41) for the T-NGS cohort, a difference of approximately three weeks. While an optimal TaT threshold was not identified for B-NGS, the ≤29-day threshold for T-NGS may be more operationally challenging given tissue processing and laboratory workflows. When used to guide concordant targeted therapy, B-NGS was associated with a survival benefit comparable to that observed with T-NGS (B-NGS HR: 1.58, 95% CI: 1.10–2.26 for empiric discordant vs. concordant therapy and T-NGS HR: 1.07, 95% CI: 0.82–1.39). These findings support the broader adoption of upfront or concurrent liquid biopsy testing as part of a comprehensive molecular profiling strategy in a/mNSCLC, particularly in settings where tissue results may be delayed, unavailable, or insufficient for NGS.

These findings highlight the critical importance of having molecular results available prior to 1L therapy initiation. Whenever feasible, treatment decisions should be guided by comprehensive molecular profiling to enable timely, guideline-concordant targeted therapy. Consistent with prior real-world studies, delays in the initiation of 1L concordant targeted therapy were associated with the suboptimal initiation of biomarker-directed therapy and compromised OS. Scott et al. demonstrated that patients who initiated 1L therapy after receiving NGS results had significantly longer OS compared with those who began empiric treatment without genomic data (mOS: 28.8 vs. 15.3 months) [14] alterations who did not receive guideline-concordant targeted therapy [6]. These findings align with our observation that delayed or absent molecular profiling contributes to inferior outcomes, even when actionable biomarkers are present.

Despite guideline recommendations advocating comprehensive molecular testing prior to treatment initiation, real-world implementation remains inconsistent. Fleming et al. found that only 20.3% of patients had core biomarker results available at their first oncology consult, with median TaTs exceeding 5 weeks [25]. Such delays often compel clinicians to initiate empiric therapy, potentially missing the therapeutic window for targeted agents. In this study, the median NGS TaT was 8 days (IQR: 7–11) for B-NGS and 28 days (IQR: 21–41) for T-NGS; however, the NGS TaT was not independently associated with the OS in either cohort. These patterns likely reflect real-world practice between 2018 and 2023, during which tissue-first testing remained standard and liquid biopsy was frequently employed as a complementary or reflex approach. As such, these findings may be less generalizable to settings where concurrent or blood-first molecular testing is routinely implemented.

This suggests that the TaT may not have an independent impact on OS when other key factors—such as biomarker status and treatment type, which have more direct effects—are taken into account. Additionally, this study did not identify an optimal cutpoint of the NGS TaT. Unexpectedly, longer TaT thresholds were associated with decreased hazards of death, which may reflect confounding due to immortal time bias limiting the interpretations. Nevertheless, this study adds real-world value by demonstrating that patients who received targeted 1L therapy had the longest median OS in both cohorts, emphasizing the survival benefit of the availability of NGS test results before commencing relevant treatment driven by findings of NGS tests. These findings support the broader adoption of rapid testing modalities, including plasma-based NGS, and institutional efforts to reduce diagnostic latency. Reasonable NGS TaTs remain crucial for maximizing the benefits of biomarker-driven therapies yet remain challenging to identify. Although TaTs might intuitively be expected to adversely affect outcomes, the absence of an independent association between the NGS TaT and OS in this study suggests that the TaT alone is unlikely to be the primary determinant of prognosis. Rather, survival differences appear to be driven by whether patients ultimately receive guideline-concordant targeted therapy once their biomarker status is known. In this context, the TaT functions as a facilitator—or barrier—to appropriate treatment selection rather than as an inherently harmful exposure. Patients with more aggressive or symptomatic disease may be unable to defer therapy, whereas those clinically stable enough to wait can benefit from biomarker-informed treatment decisions, reinforcing the importance of focusing on treatment concordance rather than TaT duration in isolation. This interpretation is consistent with this study’s time-dependent analytic framework, which was designed to capture dynamic treatment decision making rather than to evaluate TaT as a fixed baseline prognostic factor.

This study has additional limitations. The data in the FHRD are not nationally representative and there may be other factors not readily observable in this dataset (such as rapidly progressing disease) that may have required the initiation of 1L discordant non-targeted therapy before switching to a targeted agent. Furthermore, the time-dependent W1 scenarios did not adjust for multiple comparisons. A further limitation of this study is the potential for confounding by indication with respect to the timing of 1L treatment initiation. Patients who initiated 1L therapy before receiving NGS results may have done so because of more rapidly progressing or symptomatic disease, introducing systematic differences in the underlying prognoses between treatment groups that are not fully captured by the measured covariates. While the time-dependent analytic approach used in this study partially mitigates this concern by attributing follow-up time to treatment states as they occur rather than at baseline, residual confounding by unmeasured markers of disease aggressiveness, including tumor burden, rate of radiographic progression, and symptom severity at diagnosis, cannot be excluded. These factors may influence both the clinical decision to initiate empiric therapy and survival outcomes independently of biomarker status, and their absence from the analysis represents an inherent constraint of EHR-derived observational data. Future studies with more complete performance status and disease burden data would be better positioned to formally evaluate whether the survival benefit of biomarker-guided therapy is modified by baseline ECOG status. Finally, duration of follow-up was relatively short (median: 7 months), which may have limited the ability to characterize treatment patterns beyond 1L therapy. Nevertheless, this finding is consistent with real-world treatment patterns in a/mNSCLC, where approximately 40–50% do not receive 2L treatment, often due to disease progression or lack of follow-up in the health system [6,26,27].

The tertiary objective, which aimed to identify a meaningful TaT threshold, was limited by statistical constraints and by the relative homogeneity of the TaT within this study cohort. This analysis relied on dichotomization of a continuous covariate under the assumption that a biologically meaningful threshold exists; however, more than one threshold may be present, and categorization of a continuous measure may introduce confounding through loss of information and reduced statistical power. In addition, although the use of time-dependent analytic methods mitigates immortal time bias by allocating follow-up time to evolving testing and treatment states, residual immortal time and selection bias cannot be fully excluded, as patients with longer TaTs or those who ultimately received guideline-concordant therapy necessarily survived long enough to complete molecular testing and initiate treatment. This variability limits the interpretability of any single TaT cutoff point and may influence observed survival patterns independent of treatment effect. Future studies incorporating alternative modeling approaches and more complete measures of disease aggressiveness may further clarify the relationship between TaT, treatment timing, and clinical outcomes.

Collectively, our results and those of prior studies underscore the imperative to optimize molecular testing logistics. Ensuring timely access to genomic data and translating those results into appropriate 1L treatment decisions are essential for delivering guideline-concordant care and improving outcomes in a/mNSCLC.

## 5. Conclusions

This study provides evidence on the optimal use of NGS testing in managing a/mNSCLC and the importance of patients receiving upfront comprehensive molecular profiling to identify the presence or absence of any oncogenic biomarkers, enabling the receipt of appropriate biomarker-driven treatment as early as possible for patients with biomarker-driven disease. These findings can inform clinical practice guidelines to enhance personalized treatment strategies, improve patient outcomes and advance the precision oncology paradigm.

## Figures and Tables

**Table 1:** Patient demographic, clinical, and testing characteristics, overall and by NGS sample type.

		NGS Sample Type	
Patient Characteristics	Total (*n* = 4389)	Blood Only (*n* = 1806)	Tissue Only (*n* = 2583)
**Age at NGS order date (years)**			
Mean (SD)	69.9 (10.0)	70.7 (10.1)	69.3 (9.9)
Median (Q1, Q3)	71 (63, 78)	72 (64, 79)	70 (62, 77)
(Min, Max)	(29, 85)	(29, 85)	(34, 85)
**Gender,** ***n*** **(%)**			
Female	2295 (52.3)	928 (51.4)	1367 (52.9)
Male	2092 (47.7)	877 (48.6)	1215 (47.0)
Missing	2 (0.0)	1 (0.1)	1 (0.0)
**Race,** ***n*** **(%)**			
Asian	152 (3.5)	72 (4.0)	80 (3.1)
Black or African American	428 (9.8)	183 (10.1)	245 (9.5)
Other Race	365 (8.3)	117 (6.5)	248 (9.6)
White	2767 (63.0)	1169 (64.7)	1598 (61.9)
Missing	677 (15.4)	265 (14.7)	412 (16.0)
**Ethnicity,** ***n*** **(%)**			
Hispanic or Latino	156 (3.6)	62 (3.4)	94 (3.6)
Not Hispanic or Latino	4233 (96.4)	1744 (96.6)	2489 (96.4)
**ECOG at NGS order date,** ***n*** **(%)**			
0	968 (22.1)	430 (23.8)	538 (20.8)
1	1393 (31.7)	679 (37.6)	714 (27.6)
2+	754 (17.2)	378 (20.9)	376 (14.6)
Missing	1274 (29.0)	319 (17.7)	955 (37.0)
**Histology,** ***n*** **(%)**			
Non-squamous cell carcinoma	4389 (100.0)	1806 (100.0)	2583 (100.0)
**Smoking status,** ***n*** **(%)**			
History of smoking	3735 (85.1)	1519 (84.1)	2216 (85.8)
No history of smoking/not documented	654 (14.9)	287 (15.9)	367 (14.2)
**Practice type,** ***n*** **(%)**			
Academic	469 (10.7)	247 (13.7)	222 (8.6)
Community	2721 (62.0)	1497 (82.9)	1224 (47.4)
Missing	1199 (27.3)	62 (3.4)	1137 (44.0)
**Metastatic disease (stage IV) at initial diagnosis,** ***n*** **(%)**			
No	913 (20.8)	348 (19.3)	565 (21.9)
Yes	3476 (79.2)	1458 (80.7)	2018 (78.1)
**Total number of lines of therapy among treated patients**			
Mean (SD)	1.5 (0.8)	1.4 (0.8)	1.5 (0.9)
Median (Q1, Q3)	1 (1, 2)	1 (1, 2)	1 (1, 2)
(Min, Max)	(1, 7)	(1, 7)	(1, 7)
**NGS test results,** ***n*** **(%)**			
Biomarker-positive *	1692 (38.6)	682 (37.8)	1010 (39.1)
1L targeted therapy	532 (31.4)	235 (34.5)	297 (29.4)
1L non-targeted therapy	837 (49.5)	310 (45.5)	527 (52.2)
No evidence of 1L	323 (19.1)	137 (20.1)	186 (18.4)
Biomarker-negative	2460 (56.0)	1121 (62.1)	1339 (51.8)
1L targeted therapy	49 (2.0)	15 (1.3)	34 (2.5)
1L non-targeted therapy	1983 (80.6)	813 (72.5)	1170 (87.4)
No evidence of 1L	428 (17.4)	293 (26.1)	135 (10.1)
Unknown/missing	237 (5.4)	3 (0.2)	234 (9.1)
**Received 1L therapy,** ***n*** **(%)**	3401 (77.5)	1373 (76.0)	2028 (78.5)
**Received 2L therapy,** ***n*** **(%)**	1323 (30.1)	563 (31.2)	760 (29.4)
**No evidence of 1L,** ***n*** **(%)**	988 (22.5)	433 (24.0)	555 (21.5)
Died before 1L, *n* (%)	750 (17.1%)	349 (19.3%)	401 (15.5%)
**Time from advanced diagnosis to NGS specimen collection date (days)**			
Mean (SD)	9.7 (15.5)	20 (16.3)	2.5 (9.8)
Median (Q1, Q3)	0 (0, 14)	15 (8, 26)	0 (0, 0)
(Min, Max)	(0, 90)	(0, 90)	(0, 86)
**NGS test turnaround time ^†^ (imputed: capped at last confirmed activity) (days)**			
Mean (SD)	27.7 (56.9)	9.9 (32.6)	40.1 (66.2)
Median (Q1, Q3)	19 (9, 32)	8 (7, 11)	28 (21, 41)
(Min, Max)	(0, 1906)	(1, 1384)	(0, 1906)
**Time from NGS test result to 1L** **treatment initiation (days)**			
Mean (std)	25.4 (117.9)	21.7 (74.1)	27.8(140)
Median (Q1, Q3)	10 (−1, 23)	11 (2, 22)	9 (−5, 23)
(Min, Max)	(−901, 1931)	(−31, 1900)	(−901, 1931)
**Duration of follow-up from NGS** **specimen collection ^‡^ (months)**			
Mean (SD)	12.4 (14.3)	10.3 (12.5)	13.9 (15.2)
Median (Q1, Q3)	7.2 (1.9, 17.7)	5.3 (1.2, 14.6)	8.3 (2.4, 19.9)
(Min, Max)	(0.0, 73.0)	(0.0, 72.0)	(0.3, 73.0)
**Year of 1L treatment,** ***n*** **(%)**			
2018	356 (8.1)	82 (4.5)	274 (10.6)
2019	519 (11.8)	177 (9.8)	342 (13.2)
2020	589 (13.4)	221 (12.2)	368 (14.2)
2021	724 (16.5)	287 (15.9)	437 (16.9)
2022	739 (16.8)	363 (20.1)	376 (14.6)
2023	461 (10.5)	241 (13.3)	220 (8.5)
2024	13 (0.3)	2 (0.1)	11 (0.4)

The NGS test order date is the NGS specimen collection date; * biomarker-positive—EGFR, ALK, ROS1, BRAF, NTRK, KRAS G12C, RET, MET, and HER2. ^†^ The turnaround time was defined as the number of days from the date of the test order to the date of the test result. ^‡^ Follow up duration—the time from the NGS test result to the last confirmed activity date (including the NGS test result date) or the date of death, whichever occurred first; Q1, Q3—quartiles 1 and 3; *n* (%) shown unless otherwise specified. **Abbreviations**: *n*, total number; NGS, next-generation sequencing; SD, standard deviation; Q1, first quartile; Q3, third quartile; ECOG, Eastern Cooperative Oncology Group performance status; 1L, first-line therapy; Min, minimum; Max, maximum; *n* (%), number (percentage); BMI, body mass index; PDL1, programmed death-ligand 1.

**Table 2:** Adjusted overall survival multivariable Cox regression models.

Objective	Comparison	W1 Group	Number of Patients	Cohort	HR	95% CI	*p*-Value
**OS based on 1L therapy after receipt of NGS test result**	1L non-targeted discordant therapy after receipt of NGS test result with a targetable alteration vs. 1L targeted concordant therapy after receipt of NGS test result with a targetable alteration	**1e vs. 1c**	244 vs. 225	Blood	1.35	1.04–1.74	0.02
			342 vs. 270	Tissue	1.24	0.98–1.56	0.07
**OS based on timing of receipt of NGS test result**	1L non-targeted discordant therapy prior to receipt of NGS test result with a targetable alteration vs. 1L targeted concordant therapy after receipt of NGS test result with a targetable alteration	**2e vs. 1c**	64 vs. 225	Blood	1.58	1.10–2.26	0.01
			178 vs. 270	Tissue	1.07	0.82–1.39	0.61

**Abbreviations**: 1L, first-line therapy; CI, confidence interval; HR, hazard ratio; NGS, next-generation sequencing; OS, overall survival. Adjusted analyses account for the following: **W1**: time-dependent covariate controlling for NGS TaT, presence or absence of targetable alteration, time to initiation of 1L treatment, and type of 1L treatment. **LOTn**: time-dependent covariate controlling for total number of lines of therapy. **Prognostic factors**: ECOG performance status, age, smoking status, gender, body mass index (BMI), payer, race, and presence of metastatic disease at initial diagnosis. **Effect modifiers** included practice type, insurance type, socioeconomic status, time from advanced/metastatic non-small-cell lung cancer (a/mNSCLC) diagnosis to NGS test order date, and programmed death-ligand 1 (PDL1) status.

## Data Availability

Data available from corresponding author upon request.

## Abbreviations

The following abbreviations are used in this manuscript:

a/mNSCLC	Advanced/Metastatic Non-Small-Cell Lung Cancer
NSCLC	Non-Small-Cell Lung Cancer
NGS	Next-Generation Sequencing
B-NGS	Blood-Based Next-Generation Sequencing
T-NGS	Tissue-Based Next-Generation Sequencing
1. L	First-Line Therapy
OS	Overall Survival
HR	Hazard Ratio
CI	Confidence Interval
TaT	Turnaround Time
EHR	Electronic Health Record
FHRD	Flatiron Health Research Database
FDA	U.S. Food and Drug Administration
EGFR	Epidermal Growth Factor Receptor
ALK	Anaplastic Lymphoma Kinase
BRAF	B-Raf Proto-Oncogene
ROS1	ROS Proto-Oncogene 1
MET	Mesenchymal–Epithelial Transition Factor
RET	Rearranged During Transfection
NTRK	Neurotrophic Tyrosine Receptor Kinase
KRAS	Kirsten Rat Sarcoma Viral Oncogene Homolog
HER2	Human Epidermal Growth Factor Receptor 2
ERBB2	Erb-B2 Receptor Tyrosine Kinase 2
ECOG	Eastern Cooperative Oncology Group
PD-L1	Programmed Death-Ligand 1
BMI	Body Mass Index
CLL	Chronic Lymphocytic Leukemia
MCL	Mantle Cell Lymphoma
eBC	Early Breast Cancer
mBC	Metastatic Breast Cancer
SD	Standard Deviation
Q1	First Quartile
Q3	Third Quartile
Min	Minimum
Max	Maximum
LOTn	Number of Lines of Therapy

## Appendix A

### Methods

Each multivariable Cox model described in the Statistical Analysis Section of the main text also included baseline covariates representing key prognostic factors—age, gender, race/ethnicity, smoking status, body mass index (BMI), Eastern Cooperative Oncology Group (ECOG) performance status, and presence of metastatic disease at initial diagnosis—as well as potential effect modifiers (practice type [community vs. academic], insurance type, socioeconomic status, time from a/mNSCLC diagnosis to NGS order, and tumor PD-L1 status). As a sensitivity analysis, the multivariable models described above were repeated within subgroups defined by ECOG performance status (0, 1, and 2+); estimates are reported in Table A5 and Table A6 for the B-NGS and T-NGS cohorts, respectively. Within each ECOG subgroup, ECOG performance status was not included as a covariate.

**Table A1:** Molecular Alterations for Biomarkers and Associated Available Treatment.

Biomarker	Molecular Alterations [26]	Available Treatments
EGFR	Exon 19 deletions, L858R (exon 21), rare mutations (e.g., G719X, L861Q), T790M (resistance), exon 20 insertions	Afatinib, erlotinib, dacomitinib, gefitinib, osimertinib, erlotinib + ramucirumab, erlotinib + bevacizumab
ALK	Rearrangement EML4-ALK	Alectinib, brigatinib, ceritinib, crizotinib, loralatinib
BRAF	V600E mutation (exon 15)	Dabrafenib/trametinib, dabrafenib, vemurafenib
ROS1	Rearrangement CD74-ROS1 [27]	Ceritinib, crizotinib, entrectinib
NTRK	Fusions involving NTRK1, NTRK2, or NTRK3	Larotrectinib, entrectinib
MET	Exon 14 skipping mutations; gene amplification (copy number gain)	Capmatinib, crizotinib, tepotinib
RET	Rearrangement KIF5B-RET	Selpercatinib, pralsetinib
NRG1 *	Gene fusions (e.g., CD74-NRG1, SLC3A2-NRG1, SDC4-NRG1, VAMP2-NRG1, ATP1B1-NRG1) [28]	Zenocutuzumab-zbco
HER2	Activating exon 20 insertions or amplifications	Fam-trastuzumab deruxtecan-nxki
KRAS G12C	Hotspot mutations (codons 12,13,59, 61); G12C mutation	Sotorasib and adagrasib

**Abbreviations**: EGFR: Epidermal Growth Factor Receptor, includes mutations L858R (Leucine 858 Arginine, exon 21), G719X (Glycine 719 variants, e.g., G719A, G719S, G719C), L861Q (Leucine 861 Glutamine), and T790M (Threonine 790 Methionine, resistance mutation); ALK: Anaplastic Lymphoma Kinase; EML4-ALK fusions: Echinoderm Microtubule-associated Protein-Like 4 fused to ALK; BRAF: v-Raf Murine Sarcoma Viral Oncogene Homolog B; V600E: Valine 600 Glutamic acid substitution; ROS1: c-ros Oncogene 1; CD74-ROS1 rearrangements: Cluster of Differentiation 74 fused to ROS1; NTRK: Neurotrophic Tyrosine Receptor Kinase genes (NTRK1, NTRK2, NTRK3); MET: Mesenchymal–Epithelial Transition factor; RET: Rearranged during Transfection; KIF5B-RET fusions: Kinesin Family Member 5B fused to RET, activating RET kinase; NRG1: Neuregulin 1, gene fusions (e.g., CD74-NRG1 (Cluster of Differentiation 74 fused to NRG1); SLC3A2-NRG1: Solute Carrier Family 3 Member 2 fused to NRG1; SDC4-NRG1: Syndecan 4 fused to NRG1; VAMP2-NRG1: Vesicle-Associated Membrane Protein 2 fused to NRG1; ATP1B1-NRG1: ATPase Na+/K+ Transporting Subunit Beta 1 fused to NRG1; HER2: Human Epidermal Growth Factor Receptor 2 (also known as ERBB2); KRAS G12C: Kirsten Rat Sarcoma Viral Oncogene Homolog; G12C: Glycine 12 Cysteine substitution. * NRG1: only approved after progression on prior systemic therapy [5].

**Table A2:** Additional patient demographic, clinical, and testing characteristics—overall and by NGS sample type.

		NGS Sample Type	
Patient Characteristics	Total (*N* = 4389)	Blood Only (*n* = 1806)	Tissue Only (*n* = 2583)
**Region,** ***n*** **(%)**			
Midwest	678 (15.4)	275 (15.2)	403 (15.6)
Northeast	786 (17.9)	215 (11.9)	571 (22.1)
South and Puerto Rico	1679 (38.3)	838 (46.4)	841 (32.6)
West	599 (13.6)	173 (9.6)	426 (16.5)
Missing	647 (14.7)	305 (16.9)	342 (13.2)
**Insurance type,** ***n*** **(%)**			
Commercial	2743 (62.5)	1200 (66.4)	1543 (59.7)
Government sponsored	707 (16.1)	288 (15.9)	419 (16.2)
Other	182 (4.1)	79 (4.4)	103 (4.0)
Missing	757 (17.2)	239 (13.2)	518 (20.1)
**Socioeconomic status,** ***n*** **(%)**			
1—Lowest SES	727 (16.6)	327 (18.1)	400 (15.5)
2	842 (19.2)	372 (20.6)	470 (18.2)
3	849 (19.3)	352 (19.5)	497 (19.2)
4	879 (20.0)	343 (19.0)	536 (20.8)
5—Highest SES	701 (16.0)	253 (14.0)	448 (17.3)
Missing	391 (8.9)	159 (8.8)	232 (9.0)
**Practice volume at NGS order date,** ***n*** **(%)**			
Low/medium (1–200)	835 (19.0)	433 (24.0)	402 (15.6)
High (>200)	2355 (53.7)	1311 (72.6)	1044 (40.4)
Missing	1199 (27.3)	62 (3.4)	1137 (44.0)
**PDL1 status,** ***n*** **(%)**			
≥50%	910 (20.7)	275 (15.2)	635 (24.6)
1–49%	838 (19.1)	240 (13.3)	598 (23.2)
<1%	941 (21.4)	290 (16.1)	651 (25.2)
Missing or unknown	1700 (38.7)	1001 (55.4)	699 (27.1)

**Abbreviations**: NGS, next-generation sequencing; PDL1, programmed death-ligand 1; SES, socioeconomic status.

**Table A3:** Adjusted overall survival multivariable Cox regression models for additional comparators.

Covariate	B-NGS (Blood)	T-NGS (Tissue)
**W1 group (vs. 1c reference)**		
W1 = 0	0.87 (0.27–2.74)	0.96 (0.62–1.49)
1a	5.42 (4.00–7.34) ***	3.00 (2.29–3.93) ***
1b	4.61 (3.52–6.04) ***	3.92 (3.07–4.99) ***
1d	1.26 (0.58–2.74)	1.81 (1.06–3.11) *
1e	1.35 (1.04–1.74) *	1.24 (0.98–1.56)
1f	1.61 (1.29–2.01) ***	1.74 (1.42–2.15) ***
2a	NE	0.54 (0.07–3.88)
2b	1.10 (0.48–2.52)	0.80 (0.43–1.48)
2c	NE	1.90 (0.60–6.01)
2d	1.78 (0.47–6.83)	0.87 (0.54–1.39)
2e	1.58 (1.10–2.26) *	1.07 (0.82–1.39)
2f	1.79 (1.36–2.36) ***	1.74 (1.39–2.16) ***
**Prognostic factors & effect modifiers**		
Total lines of therapy (LOTn)	1.02 (1.01–1.02) ***	1.19 (1.03–1.37) *
ECOG 1 (vs. 0)	1.28 (1.09–1.50) **	1.23 (1.06–1.42) **
ECOG 2+ (vs. 0)	2.14 (1.80–2.56) ***	2.23 (1.90–2.63) ***
ECOG missing (vs. 0)	1.30 (1.07–1.58) **	1.19 (1.03–1.37) *
Age at NGS order (per year)	1.02 (1.01–1.02) ***	1.02 (1.01–1.02) ***
Metastatic at initial dx (vs. no)	1.68 (1.43–1.96) ***	1.64 (1.45–1.87) ***
No smoking history/not documented	—	0.80 (0.68–0.94) **
Gender male (vs. female)	1.34 (1.20–1.51) ***	—
Gender missing	NE	—
Practice type community (vs. academic)	1.18 (0.98–1.41)	—
Practice type missing	0.53 (0.35–0.80) **	—
Insurance government-sponsored	—	0.87 (0.76–1.00) *
Insurance other	—	0.69 (0.52–0.92) *
Insurance missing	—	0.70 (0.61–0.80) ***
SES index 2	1.05 (0.87–1.26)	1.21 (1.02–1.42) *
SES index 3	1.04 (0.85–1.25)	1.10 (0.93–1.29)
SES index 4	1.10 (0.90–1.33)	1.02 (0.86–1.20)
SES index 5 (highest)	1.11 (0.90–1.37)	0.91 (0.76–1.09)
SES index missing	1.45 (1.16–1.83) **	1.26 (1.04–1.53) *
PD-L1 TPS 1–49%	1.00 (0.80–1.25)	—
PD-L1 TPS < 1%	1.11 (0.90–1.37)	—
PD-L1 missing/unknown	1.24 (1.05–1.48) *	—
Race Black/African American	1.44 (0.96–2.15)	1.67 (1.12–2.50) *
Race other	1.87 (1.22–2.86) **	2.11 (1.42–3.13) ***
Race White	1.55 (1.07–2.24) *	1.59 (1.10–2.31) *
Race missing	1.74 (1.18–2.56) **	1.49 (1.01–2.19) *

**Abbreviations**: 1L, first-line therapy; CI, confidence interval; HR, hazard ratio; NGS, next-generation sequencing; W1—a and b are transitional and not final stages of the time-dependent variable, and patients entering these stages at certain intervals can move to other stages. Group definitions: 1a: patients with a targetable alteration as determined by NGS with no 1L; 1b: patients without a targetable alteration as determined by NGS with no 1L; 1c: 1L targeted concordant therapy after receipt of NGS test result with a targetable alteration; 1d: patients without a targetable alteration as determined by NGS prior to 1L targeted discordant therapy; 1f: patients without a targetable alteration as determined by NGS prior to 1L non-targeted concordant therapy; 2a: 1L targeted therapy and no NGS test result; 2b: 1L targeted concordant therapy prior to receipt of NGS test result with a targetable alteration; 2c: 1L targeted discordant therapy prior to receipt of NGS test result without a targetable alteration; 2d: 1L non-targeted therapy and no NGS test result; 2f: 1L non-targeted concordant therapy prior to receipt of NGS test result without a targetable alteration. Significance: * *p* < 0.05; ** *p* < 0.01; *** *p* < 0.001.

The following covariates were used to control for adjusted hazard ratios in the Cox regression models: ECOG performance status, age, metastatic disease status, smoking status, gender, practice type, BMI, insurance type, socioeconomic status, PDL1 status, and race.

**Table A4:** Independent association of NGS turnaround time with overall survival.

Objective	Comparison	Analysis Type	Number of Patients	Cohort	HR	95% CI	*p*-Value
**Independent association of TaT for NGS testing with OS**	Continuous time-dependent measure of NGS TaT, with 3-day increment	**Unadjusted**	1806	Blood	0.99	0.97–1.01	0.44
			2583	Tissue	1.00	1.00–1.00	0.97
		**Adjusted**	1806	Blood	1.00	0.98–1.02	0.97
			2583	Tissue	1.00	1.00–1.01	0.06

**Abbreviations**: CI, confidence interval; HR, hazard ratio; NGS, next-generation sequencing; OS, overall survival; TaT, turnaround time. W2: time-dependent covariate controlling for continuous NGS TaT. T2: time-dependent covariate controlling for NGS test result. T3: time-dependent covariate controlling for type of 1L treatment received. LOTn: time-dependent covariate controlling for total number of lines of therapy. Prognostic factors: ECOG performance status, age, smoking status, gender, BMI, payer, race, and presence of metastatic disease at initial diagnosis.

Effect modifiers included practice type, insurance type, socioeconomic status, time from advanced/metastatic non-small-cell lung cancer (a/mNSCLC) diagnosis to NGS test order date, and programmed death-ligand 1 (PDL1) status.

**Table A5:** Adjusted OS multivariable Cox regression by ECOG stratum—B-NGS.

Covariate	ECOG 0	ECOG 1	ECOG 2+
**W1 group (vs. 1c reference)**			
W1 = 0	0.12 (0.00–25.00)	0.76 (0.08–7.03)	1.17 (0.23–6.11)
1a	4.46 (2.15–9.25) ***	8.17 (4.86–13.73) ***	4.01 (2.32–6.91) ***
1b	2.83 (1.60–5.01) ***	5.42 (3.34–8.80) ***	3.70 (2.29–6.00) ***
1d	1.21 (0.28–5.11)	4.59 (1.39–15.19) *	0.94 (0.22–3.97)
1e	1.08 (0.65–1.80)	1.51 (0.98–2.33)	1.16 (0.64–2.10)
1f	1.32 (0.86–2.03)	1.72 (1.18–2.53) **	1.91 (1.21–3.01) **
2a	NE	NE	NE
2b	1.46 (0.44–4.83)	1.31 (0.40–4.32)	NE
2c	—	—	—
2d	NE	NE	3.10 (0.33–29.34)
2e	0.99 (0.46–2.11)	2.16 (1.20–3.89) *	2.75 (1.30–5.81) **
2f	1.70 (0.98–2.95)	2.10 (1.34–3.29) **	1.85 (1.03–3.31) *
**Prognostic factors & effect modifiers**			
Total lines of therapy (LOTn)	1.38 (1.17–1.63) ***	1.58 (1.32–1.89) ***	—
Age at NGS order (per year)	1.03 (1.01–1.04) ***	1.02 (1.01–1.03) **	—
Metastatic at initial dx (vs. no)	1.68 (1.24–2.28) **	1.44 (1.12–1.86) **	1.71 (1.24–2.36) **
No smoking history/not documented	—	—	—
Gender male (vs. female)	—	1.42 (1.17–1.73) ***	1.64 (1.30–2.07) ***
Race Black/African American	—	2.64 (1.31–5.31) **	—
Race other	—	3.95 (1.89–8.24) ***	—
Race White	—	2.43 (1.27–4.63) **	—
Race missing	—	3.68 (1.85–7.30) ***	—

Hazard ratio (95% CI). Significance: * *p* < 0.05; ** *p* < 0.01; *** *p* < 0.001. NE: not estimable. Models stratified by ECOG performance status (0, 1, 2+).

**Table A6:** Adjusted OS multivariable Cox regression by ECOG stratum—T-NGS (tissue).

Covariate	ECOG 0	ECOG 1	ECOG 2+
**W1 group (vs. 1c reference)**			
W1 = 0	1.28 (0.23–7.23)	0.44 (0.12–1.61)	2.44 (1.06–5.60) *
1a	7.11 (3.15–16.07) ***	3.90 (2.36–6.47) ***	5.26 (2.81–9.87) ***
1b	12.33 (6.04–25.15) ***	5.12 (3.24–8.09) ***	6.44 (3.67–11.31) ***
1d	3.10 (0.69–13.99)	2.80 (0.98–8.02)	3.19 (0.95–10.78)
1e	2.89 (1.53–5.46) **	1.32 (0.88–1.98)	1.29 (0.72–2.29)
1f	4.37 (2.41–7.90) ***	1.77 (1.22–2.57) **	1.55 (0.95–2.52)
2a	NE	0.93 (0.12–7.28)	NE
2b	2.92 (0.65–13.14)	0.47 (0.11–1.97)	0.19 (0.03–1.44)
2c	—	2.84 (0.68–11.90)	—
2d	1.80 (0.50–6.51)	0.73 (0.30–1.77)	1.49 (0.62–3.58)
2e	2.30 (1.17–4.51) *	0.95 (0.60–1.50)	1.58 (0.84–2.95)
2f	4.81 (2.63–8.80) ***	1.45 (0.97–2.15)	1.92 (1.16–3.16) *
**Prognostic factors & effect modifiers**			
Total lines of therapy (LOTn)	0.89 (0.67–1.17)	2.13 (0.94–4.84)	NE
Age at NGS order (per year)	1.02 (1.00–1.03) **	1.02 (1.01–1.03) **	—
Metastatic at initial dx (vs. no)	1.81 (1.39–2.35) ***	1.54 (1.22–1.94) ***	1.55 (1.07–2.23) *
BMI underweight (<18.5)	0.53 (0.30–0.94) *	—	—
BMI overweight (25–<30)	0.89 (0.67–1.17)	—	—
BMI obese (≥30)	1.02 (0.76–1.39)	—	—
BMI missing	2.75 (1.53–4.94) ***	—	—
PD-L1 TPS 1–49%	—	1.08 (0.83–1.41)	—
PD-L1 TPS < 1%	—	1.44 (1.11–1.86) **	—
PD-L1 missing/unknown	—	1.31 (1.00–1.70) *	—
Race Black/African American	—	2.13 (0.94–4.84)	—
Race other	—	2.82 (1.26–6.32) *	—
Race White	—	2.17 (1.01–4.67) *	—
Race missing	—	1.68 (0.76–3.74)	—

Significance: * *p* < 0.05; ** *p* < 0.01; *** *p* < 0.001.
